# Antibacterial efficacy of a chitosan-based hydrogel loaded with epsilon poly-L-lysine and poly(I:C) extracellular vesicles for the control of polymicrobial extremity wound infections in a porcine polytrauma model

**DOI:** 10.1016/j.mmr.2026.100011

**Published:** 2026-03-23

**Authors:** Cole E. Ogrydziak, Catherine F.T. Uyehara, Lee-Ann M. Murata, Wendy E. Kurata, Lauren N. Wong, Randal S. Dudis, Emily J. Bailey, Lisa M. Pierce

**Affiliations:** Department of General Surgery, Tripler Army Medical Center, Hawaii 96859, USA; Department of Clinical Investigation, Tripler Army Medical Center, Hawaii 96859, USA

**Keywords:** Chitosan hydrogel, Epsilon poly-L-lysine, Extracellular vesicles, Polymicrobial-infected extremity soft tissue injury, *Acinetobacter baumannii*, *Escherichia coli*, *Staphylococcus epidermidis*, Porcine polytrauma model, Pulmonary contusion, Hemorrhage

Dear Editor,

Traumatic wound infection is a major risk to the full recovery of the warfighter in the current and future operational environment [1]. Extremity injuries comprise the majority of combat wounds, with a significant proportion (53%) of extremity injuries involving penetrating soft-tissue wounds [2]. Combat wounds are contaminated typically from debris in explosive devices, uniforms, and soil, which may lead to infection and possibly higher mortality rates if trauma care is delayed under austere conditions when medical evacuation is not immediately feasible [1]. Conventional treatment includes bandaging wounds with sterile dressings, stabilizing fractures, administering antibiotics, and transferring to surgical support as soon as feasible [1]. The urgency for novel, non-antibiotic approaches to control and eliminate multidrug-resistant (MDR), biofilm-forming bacteria is highlighted by the fact that antibiotic resistance is increasing globally and was reported as the third leading cause of death worldwide in 2019 [1], [3]. Medical technology development directed against MDR organisms and wound infection in the setting of prolonged care and potentially strained medical logistics support is, therefore, a top priority for the military [1].

The hyperinflammatory systemic and local response to trauma may influence wound progression, and wound healing also may be impaired by local and systemic inflammatory responses secondary to bacterial colonization [4]. Because the inflammatory state and immune function at the time of traumatic injury may have a confounding effect on wound healing and biofilm formation, it is important to investigate novel therapeutics for the treatment of combat wound infections under the typical conditions of blood loss and traumatic changes in tissue perfusion and oxygenation. We previously found that a chitosan (CS)-based hydrogel containing the broad-spectrum antimicrobial peptide epsilon poly-L-lysine (EPL) demonstrated significant antibiofilm activity when applied topically to a porcine ex vivo skin wound polymicrobial biofilm model containing MDR *Pseudomonas aeruginosa* (*P. aeruginosa*), methicillin-resistant *Staphylococcus aureus*, and *Candida albicans* [5]. We also showed that the CS/EPL hydrogel demonstrated low cytotoxicity and reduced established *P. aeruginosa* biofilms in an *in vivo* mouse model of infected burn wounds [6]. Additionally, we discovered that human mesenchymal stem cell (MSC)-derived extracellular vesicles (EVs) contained proteins important in innate immunity and host defense whose levels were increased by priming parental MSCs with the Toll-like receptor 3 agonist polyinosinic-polycytidylic acid [poly(I:C)] [7]. Therefore, in the current study we investigated the *in vivo* antibacterial efficacy of a promising injectable CS hydrogel modified with EPL and poly(I:C) EVs to control polymicrobial traumatic extremity wound infection in the acute period after injury using a porcine polytrauma model incorporating pulmonary contusion, hemorrhage, liver laceration, and endotoxin-induced systemic inflammation. The primary aim of this study was to validate the efficacy of the CS/EPL hydrogel in a clinically relevant large animal model under the conditions of severe polytrauma and physiological stress. A powerful antimicrobial, hemostatic hydrogel that can be injected directly into deep soft-tissue wounds early in the clinical course to reduce infection risks would provide a simple tool for providers in prehospital environments that would require minimal medical training.

Nine anesthetized, mechanically ventilated Duroc-cross pigs underwent polytrauma consisting of blunt force lung contusion, liver laceration+class III–IV hemorrhage, endotoxin administration, and polymicrobial (*Acinetobacter baumannii*, *Escherichia coli*, and *Staphylococcus epidermidis*)-infected extremity soft tissue injury (4 hind leg wounds/pig) (**Additional file 1: Methods and** Fig. S1). After 1 h inoculation, each wound was treated with sterile saline-soaked gauze or hydrogel (CS, CS/EPL, or CS/EPL/EVs) injected to fill the wound cavity (**Additional file 1:** Fig. S2). All 4 treatments were included in 8 polytrauma pigs, while 1 pig had wounds treated with only saline-soaked gauze or CS/EPL and therefore was not included in the bacterial load analyses. Wound biopsies were assessed for bacterial content by conventional plating after 5 h of treatment. Results were compared to 5 control pigs that underwent wound infection without polytrauma.

Poly(I:C) EV characterization is presented in **Additional file 1:** Fig. S3**,** Table S1**, and Additional file 2**. Polytrauma pigs developed macroscopic lung damage, pulmonary hypertension as indicated by increased pulmonary vascular resistance to systemic vascular resistance ratio (*P*<0.001), sustained hypotension (decreased mean arterial pressure, *P*<0.001), and elevated systemic inflammatory cytokines interleukin (IL)-6 (*P*<0.001), IL-1 receptor antagonist (IL-1ra) (*P*<0.001), and IL-18 (*P*=0.001) (**Additional file 1:** Figs. S4-S5**, and Additional file 3**). These findings were consistent with physiological responses to trauma and hemorrhage commonly observed in humans [8]. Wounds treated with CS/EPL and CS/EPL/EVs for 5 h in the setting of polytrauma had a 4-log reduction in viable bacteria vs. saline-soaked gauze (*P<*0.001), which was not inferior to efficacy in control pigs (Fig. 1). No further benefit was noted with the addition of EVs (CS/EPL vs. CS/EPL/EVs, *P*=1.000). Interestingly, a 2.6-log increase in bacterial load was observed in polytrauma wounds treated with saline-soaked gauze compared to those without polytrauma (*P<*0.001) (Fig. 1). This may be indicative of the increased inflammatory state and relative immunocompromise associated with trauma, and emphasizes the need for good antibacterial strategies.

**Fig. 1:**
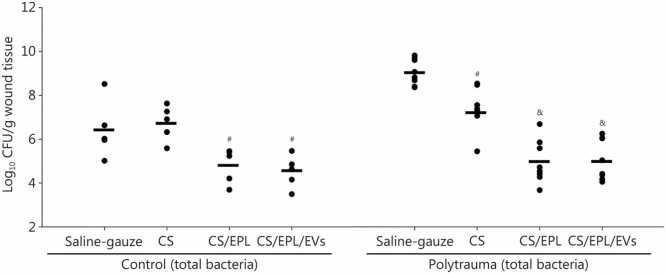
Bacterial load recovered from treated wounds in control pigs (*n*=5) and polytrauma pigs (*n*=8). Each animal had 4 extremity wounds and received all 4 treatments. ^#^*P<*0.05 vs. saline-soaked gauze, ^&^*P<*0.05 vs. saline-soaked gauze and CS, one-way repeated measures ANOVA followed by post hoc multiple comparisons using a Bonferroni *t*-test. Bar indicates mean value. CFU. Colony forming units; CS. Chitosan hydrogel; CS/EPL. Chitosan/epsilon poly-L-lysine hydrogel; CS/EPL/EVs. Chitosan/epsilon poly-L-lysine/extracellular vesicles hydrogel.

Therefore, we found that an easily injectable CS-based hydrogel loaded with EPL demonstrated efficacy to control polymicrobial traumatic extremity wound infections *in vivo* in a comprehensive porcine model of polytrauma designed to mimic the complex injuries and physiological responses observed in combat casualties. Multifunctional hydrogels incorporating CS and EPL as the major components using different formulations to improve their mechanical, hemostatic, and antimicrobial properties have recently gained increasing interest for deep tissue hemostasis and infected wound healing [9].

We were unable to show an increased benefit of EVs released from poly(I:C)-activated human MSCs incorporated into the CS/EPL hydrogels during the acute observation period in this study. The beneficial effects of EVs are mostly applied indirectly through the reprogramming of immune cells and the activation of innate and adaptive immune responses rather than direct bactericidal activity, and it is conceivable that the effects of EV-loaded hydrogels on immunomodulation of the dysregulated hemorrhagic shock-induced innate immune response and tissue damage at the site of injury may not be observed until day(s) after treatment [10]. It is also possible that hemorrhagic shock, systemic inflammation, or endotoxemia in the polytrauma model may blunt EV-mediated immunological responses, and the slow-release kinetics of EVs from the hydrogels do not align with the 5 h endpoint. Therefore, the lack of effect should not be interpreted as biological inactivity of EVs, but rather as a limitation of the acute experimental design. Future studies with longer durations of treatment are needed to evaluate the long-term antimicrobial and wound healing benefits of poly(I:C) EVs.

Limitations of this study include the relatively small control group (*n* = 5) and the use of multiple wounds per animal, which increases the potential for within animal clustering. The study was exploratory in nature, and no a priori power calculation was performed. In addition, mechanistic studies at the wound site were not performed. Bloodstream infections occurred in one polytrauma animal and in one control animal in this study, and in both cases, isolates were identified as *Acinetobacter baumannii.* However, it was not possible to determine which specific wound or wound treatment was the source of the systemic *Acinetobacter baumannii.*

In conclusion, a CS hydrogel containing EPL showed efficacy to control polymicrobial wound infection *in vivo* in an established porcine polytrauma model. Poly(I:C) EVs did not enhance the antibacterial efficacy of CS/EPL in the acute period. This easily injectable, biocompatible hydrogel shows promise as a novel alternative to antibiotics to treat traumatic wound infections at the point of injury that may be used in far-forward environments to improve outcomes in the injured warfighter. Additional studies evaluating the addition of a hemostatic agent(s) to increase the hydrogel’s inherent hemostatic properties, the local inflammatory response to the hydrogel at the wound site, and the long-term effects of the hydrogel on wound healing are warranted.

## 1 Ethics approval and consent to participate

The study protocol was approved by the Institutional Animal Care and Use Committee at Tripler Army Medical Center (TAMC23A45) and was performed in strict accordance with ARRIVE guidelines. Investigators complied with policies as prescribed in the U.S. Department of Agriculture Animal Welfare Act and the National Research Council’s Guide for the Care and Use of Laboratory Animals. Facilities are fully accredited by the Association for Assessment and Accreditation of Laboratory Animal Care International.

## Funding

This work was supported by internal funds from the Department of Clinical Investigation, Tripler Army Medical Center.

## Data Availability

All data generated or analyzed during this study are included in the published article and its supplementary information file. Further inquiries are available from the corresponding author upon reasonable request.

## Appendix A Supplementary material

Supplementary material**Additional file 1. Methods**. **Fig. S1** Overview of the *in vivo* experimental timeline and procedures during establishment of the polytrauma state. **Fig. S2** Representative images of the CS/EPL hydrogel and its application in the extremity wound cavity in the porcine polytrauma model. **Fig. S3** Poly(I:C) EV characterization. **Fig. S4** Hemodynamics and plasma creatinine levels in polytrauma and control pigs, including mean arterial pressure, cardiac output, PVR/SVR ratio, and plasma creatinine. **Fig. S5** Elevation of systemic inflammatory cytokine (IL-6, IL-1ra, and IL-18) levels in polytrauma. **Table S1** Release kinetics of EVs from hydrogels

Supplementary material**Additional file 2.** Proteins detected in poly(I:C) EV samples with high confidence

Supplementary material**Additional file 3.** Data from polytrauma and control pigs
