# Supplementary material for: Antibacterial efficacy of a chitosan-based hydrogel loaded with epsilon poly-L-lysine and poly(I:C) extracellular vesicles for the control of polymicrobial extremity wound infections in a porcine polytrauma model
Source: Mil Med Res. 2026 Mar 23;13(1):100011. doi: 10.1016/j.mmr.2026.100011 (PMC13054572; doi:10.1016/j.mmr.2026.100011)
Supplement: Supplementary file 1 — Supplementary materialAdditional file 1. Methods. Fig. S1 Overview of the in vivo experimental timeline and procedures during establishment of the polytrauma state. Fig. S2 Representative images of the CS/EPL hydrogel and its application in the extremity wound cavity in the porcine polytrauma model. Fig. S3 Poly(I:C) EV characterization. Fig. S4 Hemodynamics and plasma creatinine levels in polytrauma and control pigs, including mean arterial pressure, cardiac output, PVR/SVR ratio, and plasma creatinine. Fig. S5 Elevation of systemic inflammatory cytokine (IL-6, IL-1ra, and IL-18) levels in polytrauma. Table S1 Release kinetics of EVs from hydrogels [file mmc1.pdf]

Methods

Cell culture and priming

Using a Quantum bioreactor (Terumbo BCT, Inc., Lakewood, Colorado, USA), human bone marrow-derived mesenchymal stem cells (MSCs) purchased from RoosterBio, Inc. (Frederick, Maryland, USA) were seeded at approximately 20 million cells and were grown in RoosterBio media per the manufacturer's instructions. After 5 d, to switch MSCs towards a more immunoregulatory phenotype, toll-like receptor 3 agonist polyinosinic-polycytidylic acid [poly(I:C)] at 1 μg/ml (MilliporeSigma, Burlington, Massachusetts, USA) was added to fresh media, and MSCs were treated for 1 h [1-4]. Media was then changed to a 1:1 mixture of Dulbecco's Modified Eagle Medium (DMEM)+5% Exo-FBS (exosome-deficient fetal bovine serum; System Biosciences, Palo Alto, California, USA) and RoosterCollect reagent. Extracellular vesicle (EV) media were harvested in 2×250 ml collections (at 48 and 72 h) and were frozen at –80 ºC until EV isolation.

EV isolation

Cell debris was removed from thawed EV media by centrifugation at 300×*g* for 10 min and then 3000×*g* for 30 min. Media was passed through a 0.22 μm filter, and then EVs were isolated using tangential flow filtration (TFF) using a Minimate EVO System with a 300 kDa Minimate TFF capsule (Pall Corporation, Port Washington, New York, USA). Each batch of EV media (approximately 500 ml) was concentrated, and buffer exchanged using TFF and further concentrated using a 10 kD molecular weight Amicon Ultra-15 Centrifugal Filter (MilliporeSigma). Aliquots of EVs were diluted in sterile phosphate-buffered saline (PBS) and were frozen at –80 ºC until use.

EV characterization

EV size and concentration were determined using nanoparticle tracking analysis by means of a NanoSight NS300 (Malvern Panalytical Inc., Westborough, Massachusetts, USA). Total protein concentration of EVs was determined using the bicinchoninic acid (BCA) protein assay (ThermoFisher Scientific, Waltham, Massachusetts, USA) following the manufacturer's

instructions. For morphological analysis of EVs, transmission electron microscopy using a Hitachi HT-7700 microscope at 80 kV (Hitachi High-Technologies Corp., Tokyo, Japan) was performed as a service by Creative Bioarray (Shirley, New York, USA). Western blotting analysis was performed as a service by RoosterBio for measurement of exosomal protein markers cluster of differentiation (CD) 9, CD63, and CD81 using primary antibodies at 1:1000 dilution and goat anti-mouse secondary antibodies at 1:20,000 dilution (ThermoFisher). Membranes were developed using SuperSignal West Pico Plus Chemiluminescent Substrate (ThermoFisher) and imaged using a C-Digit Blot Scanner (LI-COR Inc., Lincoln, Nebraska, USA).

Proteomic analysis

The protein content of two poly(I:C) EV samples isolated from different batches used to treat the pigs in this study underwent mass spectrometry performed by Creative Bioarray using their Label-Free Based Proteomics Analysis Service. Briefly, EV protein samples were digested with trypsin, and peptides were identified/quantified by applying Creative Bioarray's nano-LC-MS/MS (liquid chromatography-tandem mass spectrometry) platform. Peptide signatures were analyzed and searched against a human protein database using the proteomics software package MaxQuant version 1.6.2.6 (www.maxquant.org). Only peptides identified with high confidence were chosen for downstream protein identification analysis. Identified proteins were analyzed for enrichment in terms of their Gene Ontology (GO) categories using the Functional Annotation Tool in the Database for Annotation, Visualization, and Integrated Discovery (DAVID Bioinformatics Resource) using default settings [5,6]. Enrichment analysis in DAVID used a modified Fisher Exact test to determine whether proteins were enriched in the annotation categories, and enrichment statistics were adjusted for multiple hypothesis testing by the Benjamini correction.

Preparation of chitosan (CS)/epsilon-poly-L-lysine (EPL)/ Poly(I:C) EVs hydrogels

Thermosensitive CS hydrogels using β-glycerophosphate (β-GP) as a gelling agent were made using CS possessing a high degree of deacetylation (95.6%) and low viscosity (66 centipoise; ChitoLytic

Inc., Ajax, Ontario, Canada) [7,8]. A 3.56% (weight/volume) CS solution was prepared in 1% (volume/volume) acetic acid (MilliporeSigma), autoclaved at 121 °C for 15 min, and stored at 4 °C until hydrogel production. EPL (epsilon-polylysine; AA Blocks Inc., San Diego, California, USA) was prepared as a 28% (weight/volume) stock solution in sterile Dulbecco's PBS (DPBS; MilliporeSigma) and was added to the CS solution prior to the addition of β-GP. The final concentration of EPL in the hydrogels was 1%. β-glycerophosphate disodium salt pentahydrate (1 g/ml in DPBS; MilliporeSigma) was added dropwise to the CS solution in a 5.5:1 (CS:β-GP) volume ratio to obtain a final concentration of 2.54% CS and 14.3% β-GP. After mixing in poly(I:C) EVs (approximately 2.5×10^{10} EVs or 200 µg EV protein per ml hydrogel), the solution was pre-loaded into syringes and placed in an incubator at 37 °C for 1 h to enable the thermo-irreversible gelation process. The chosen dose is in accordance with effective EV doses used in published studies [9,10]. Additional CS and CS/EPL hydrogels were prepared by replacing the volume of EPL and/or EVs with an equal volume of DPBS. The pH of the hydrogels was approximately 7.0. Hydrogels were stored at 4 °C until use (within one week). Characterization of CS/EPL hydrogels (water uptake and in vitro degradation analyses, cytotoxicity, EPL release, and antibacterial activity) was reported previously [7,8].

EV release from hydrogels

To evaluate the release kinetics of EVs from hydrogels, 275 µl CS solution incorporating 30 µg poly(I:C) EVs was loaded into the upper chamber of each Transwell (6.5 mm Transwell with 5.0 µm pore polycarbonate membrane insert; MilliporeSigma) in 24-well plates and incubated at 37 °C to form gels as described above. DPBS (1.5 ml) was added to each well, and plates were incubated at 37 °C for up to 7 d. Supernatant was collected from CS/EVs and CS hydrogels (n=3 each per time point) at 2, 5, 24, 48, 96, and 168 h and stored at –80 °C until analysis. Protein concentration was determined in supernatants using the micro BCA protein assay kit (ThermoFisher Scientific), assayed in triplicate to generate an EV release curve.

Bacterial strains and growth conditions

Escherichia coli (*E. coli*, 25922), *Acinetobacter baumannii* (*A. baumannii*, BAA-747), and *Staphylococcus epidermidis* (*S. epidermidis*, 12228) were purchased from American Type Culture Collection (Manassas, Virginia, USA). These strains were chosen because most of the combat-related extremity wound infections among servicemembers were polymicrobial, and a high prevalence of Gram-negative *E. coli* and *A. baumannii* strains were isolated from these combat casualties [11]. *S. epidermidis* is a Gram-positive bacterium that is one of the most abundant colonizers of human skin, is known to form biofilms on implanted medical devices, and intraoperative cultures in patients experiencing gunshot wounds to the foot and ankle yielded *S. epiderimidis* in 26% of cases [12].

Prior to experimental testing, bacterial stocks stored at –80 ^{o}C on cryobeads were serially passed twice on blood agar plates and incubated at 37 ^{o}C to produce viable colonies. Isolated bacterial colonies were inoculated in tryptic soy broth, adjusted to 0.5 McFarland, and grown overnight at 37 ^{o}C with shaking at 225 rpm. Cultures were diluted 1:100 in tryptic soy broth [approximately 1×10^{7} colony forming units (CFU)/ml], combined at a volume ratio of 1:1:1 (*E. coli*:*A. baumannii*:*S. epidermidis*), and then incubated with metal discs (2 cm diameter, 0.2 mm thick) for 5 h at 37 ^{o}C with shaking at 150 rpm. Each porcine leg wound was inoculated with a metal disc for 1 h before the disc was removed and wound treatment began. A subset of discs recovered from porcine wounds was inoculated on blood agar plates at 37 ^{o}C for 48 h, revealing growth of all 3 bacterial strains.

Porcine polytrauma model, *in vivo* study design, and experimental timeline

The study protocol was approved by the Institutional Animal Care and Use Committee at Tripler Army Medical Center (TAMC23A45). Investigators complied with policies as prescribed in the U.S. Department of Agriculture Animal Welfare Act and the National Research Council's Guide for the Care and Use of Laboratory Animals. Facilities are fully accredited by the Association for Assessment and Accreditation of Laboratory Animal Care International. A total of 17 Duroc-cross

pigs were used, including 14 healthy experimental pigs used in analysis [(26.7±0.8) kg; 9 males, 5 females] and 3 pigs that were excluded from analysis (1 model development, 1 gastrointestinal health issues, and 1 ventilator-associated complications). Nine polytrauma (PT) pigs were subjected to multiple injuries in the following order: blunt chest trauma, polymicrobial-infected extremity soft tissue injury, penetrating abdominal injury (liver laceration-induced uncontrolled hemorrhage), followed immediately by controlled hemorrhage, and low-dose endotoxin administration (**Additional file 1: Fig. S1**). Five control pigs underwent extremity wound infection without PT. After 1 h of inoculation, extremity wounds were treated for 5 h, and then animals were euthanized by infusion of a pentobarbital-based euthanasia solution (approximately 6 h after trauma initiation). All experimental procedures were conducted under deep anesthesia. Hemodynamic and blood gas measurements were monitored throughout the experiment. Blood samples were taken at baseline (P1) and at each period of establishment of the PT state including 1.5 h after pulmonary contusion and extremity soft tissue injury (P2), 1 h after liver laceration and hemorrhage (P3), approximately 1.5 h after endotoxin administration (P4), and after 5 h of extremity wound treatment (P5) for assessment of tissue oxygenation and systemic inflammatory markers. Wound biopsies collected at euthanasia were assessed for bacterial content by conventional plating, and blood samples were cultured on blood agar at 37 °C for 48 h to determine the presence of bloodstream infections.

Anesthesia, instrumentation, and measurements

Pigs underwent ≥72 h of acclimation prior to experimentation. Pigs were fasted overnight with *ad lib* water. Before surgery, pigs were premedicated with acepromazine (1.1 mg/kg), ketamine (33 mg/kg), and atropine (0.04 mg/kg) administered intramuscularly. Animals underwent endotracheal intubation and were mechanically ventilated (Servo-i; Maquet Medical Systems, Wayne, New Jersey, USA) to maintain tidal volumes of approximately 8 ml/kg, positive end-expiratory pressure of 5 mmHg, and end-tidal CO_2 of 35–45 mmHg. The fraction of inspired oxygen (FiO_2) was maintained at 21% throughout most of the experiment, but was increased if needed to recover the animal to survive the 6 h of post-trauma stabilization. Anesthesia was maintained with ketamine (8–

33 mg/kg per hour), fentanyl (5–25 μg/kg per hour), and midazolam (0.5–1.5 mg/kg per hour), and infusion rates were titrated to maintain adequate sedation. A bolus of 10 ml/kg normal saline was given during instrumentation to correct for interindividual differences in preoperative fluid balance. Normal saline was administered continuously throughout the experiment to maintain hydration and was adjusted based on titration of anesthetics to maintain the total fluid administration rate at 6 ml/kg per hour.

Pigs were surgically instrumented with saline-filled catheters for hemodynamic monitoring, blood draws, and infusions. A 7F 20-cm introducer sheath (Teleflex, Inc., Morrisville, North Carolina, USA) or 1/16-inch ID Flexelene tubing (Eldon James, Loveland, Colorado, USA) was placed in the abdominal aorta via the right femoral artery for hemorrhage. A 7F 16-cm triple-lumen catheter (Arrow Three-Lumen CVC; Teleflex, Inc., Morrisville, North Carolina, USA) was inserted into the left femoral vein for administration of fluids and medications. A 4F 16-cm arterial PiCCO (pulse contour cardiac output; Getinge, Gothenburg, Sweden) catheter was placed into the left femoral artery for mean arterial blood pressure (MAP) and thermodilution continuous cardiac output (CO) monitoring. Two 5F 8-cm double-lumen catheters (Small Vein Arrowg+ard Blue Two-Lumen CVC; Teleflex, Inc., Morrisville, North Carolina, USA) were placed cephalad into the left carotid artery and right external jugular vein for measurement of blood gases and blood pressure. A 7F 110-cm pulmonary artery catheter (Swan-Ganz; Edwards Lifesciences, Irvine, California, USA) was inserted through the right external jugular vein with a 9F introducer sheath (Cook, Inc., Bloomington, Indiana, USA) to measure pulmonary artery pressure, mixed venous oxygen saturation, and core temperature. A Foley catheter (Rusch Teleflex Medical, Research Triangle Park, North Carolina, USA) was placed into the bladder via midline laparotomy for urine collection.

All pressure monitoring catheters were connected to transducers and zeroed to the level of the animal’s mid-heart. Placement of catheters was verified by the observation of characteristic wave forms. A standard lead II electrocardiogram (IntelliVue MP70; Philips Medical Systems, Andover, Massachusetts, USA) was used to monitor cardiac rhythm. Body core temperature was maintained

with a heating blanket (Bair Hugger; 3M, St. Paul, Minnesota, USA). Blood gases were measured with a blood gas analyzer (ABL 800; Radiometer America, Inc., Westlake, Ohio, USA). Oxygen consumption was measured by respirometry (Vmax Encore, Viasys Healthcare, Conshohocken, Pennsylvania, USA).

Induction of polytrauma

After achieving stable baseline conditions (approximately 1 h after instrumentation), blunt chest trauma was induced by firing a MasterShot 0.22 caliber powder-actuated tool (Ramset, Glenview, Illinois, USA) 3 times against a 10-cm square (1 cm thick) aluminum plate applied to the right thorax at the mid to anterior axillary line. Next, 4 soft tissue wounds (approximately 2.5 cm in length and 2.5 cm in depth) were created in the hind legs using a scalpel (two wounds per thigh region at least 3–4 cm apart). A 2 cm metal disc contaminated with *E. coli*, *A. baumannii*, and *S. epidermidis* (described above) was inserted into each wound. Wounds were covered with Tegaderm (3M Health Care, St. Paul, Minnesota, USA) and allowed to inoculate for 1 h before disc removal and treatment with either 1) sterile saline-soaked gauze, 2) CS, 3) CS/EPL, or 4) CS/EPL/poly(I:C) EVs hydrogel injected into the wound cavity (2 ml hydrogel per wound). Each pig received all treatments, thereby serving as its own control.

After treatment of extremity wounds, a midline laparotomy was performed, followed by liver laceration with two 3 cm cross-like incisions through one third of liver tissue using a scalpel. Thirty seconds of uncontrolled bleeding was allowed before using a tamponade consisting of 4 sterile packs of 10 cm×10 cm gauze [13]. Volume of blood loss from the liver was estimated by weighing the blood-soaked gauze and was included in the estimation of total blood loss during hemorrhage. At the time of liver laceration, pressure-controlled and volume-limited hemorrhagic shock was induced by arterial blood withdrawal at a rapid rate of 3 ml/kg per min for 7 min followed by 1 ml/kg per min until reaching a MAP drop of 50% from baseline (to 35–45 mmHg) or a maximum withdrawal of 50% of estimated total blood volume of 60 ml/kg. Hemorrhage was paused if MAP dropped below 35 mmHg. This resulted in an initial 50% drop in MAP within 10 min of bleeding and a total

blood loss of 21–30 ml/kg (class III–IV hemorrhage) within 30 min. Animals then underwent an additional 30-minute period where no intervention or resuscitation was performed.

After achieving a stable state of sustained hypotension (minimum 30% decrease in MAP) 1 h after the initiation of liver injury+hemorrhage, a low dose of endotoxin (100 ng in 1 ml normal saline; lipopolysaccharide from *E. coli* O55:B5; MilliporeSigma) was administered intravenously in 25 ng increments over a 15-minute period. This direct injection of low-dose endotoxin into the systemic circulation was designed to hasten the achievement of the target inflammatory state induced by PT and wound infection in the acute observation time used in this study [14]. Subsequent to the endotoxin injection, intermittent administration of supplemental oxygen (FiO_2 up to 40%) and less than one shed volume of whole blood and/or normal saline occurred if needed to ensure survivability during the stabilization period.

Multiplex cytokine analysis

The Milliplex Porcine Cytokine/Chemokine Magnetic Bead Panel (MilliporeSigma, Burlington, Massachusetts, USA) was used with the Luminex analyzer MAGPIX (Diasorin, Stillwater, Minnesota, USA) to measure circulating inflammatory cytokine levels in blood plasma samples using a standard curve following the manufacturer's instructions.

Quantitation of viable bacteria recovered from polymicrobial-infected extremity wounds

For CFU determination, wound biopsies (approximately 1 g) were placed in 10 ml sterile saline, vortexed for 1 min, and sonicated for 5 min in a Bransonic CPX2800H ultrasonic bath (40 kHz; Branson Ultrasonics Corp., Danbury, Connecticut, USA). Serial dilutions (10^1 to 10^{-7}) were plated onto blood agar for measurement of total bacteria. Plates were grown for 36–48 h at 37 ºC before counting colonies. The lower limit of detection was 1×10^2 CFU. CFU were normalized per g of wound tissue. Additional selective agar plates, including mannitol salt agar and MacConkey agar, were used to distinguish Gram-positive *S. epidermidis* and to differentiate Gram-negative *E. coli* and *A. baumannii*, respectively.

Statistical analysis

Results were reported as means±standard error of the mean (SEM). A two-way repeated measures analysis of variance (ANOVA) followed by post hoc multiple comparisons using a Bonferroni *t*-test was used to determine differences in hemodynamics and inflammatory cytokine levels between treatment groups (PT and control) at the various periods [baseline, after pulmonary contusion and extremity soft tissue injury (trauma), after liver laceration and hemorrhage (shock), after endotoxin administration (inflammation), and after 5 h of extremity wound treatment (stabilization)]. A one-way repeated measures ANOVA followed by post hoc multiple comparisons using the Bonferroni *t*-test was used to compare CFU counts among the 4 wound treatments in each pig for the PT and control groups. A *t*-test was used to compare bacterial load between PT and control groups in wounds treated with saline-soaked gauze. Statistical analyses were performed using SigmaPlot 15.0 software (Grafiti LLC, Palo Alto, California, USA) with a P-value<0.05 considered significant.

References

1. Pierce LM, Kurata WE. Priming with toll-like receptor 3 agonist poly(I:C) enhances content of innate immune defense proteins but not microRNAs in human mesenchymal stem cell-derived extracellular vesicles. Front Cell Dev Biol. 2021;9:676356.
2. Monsel A, Zhu YG, Gennai S, Hao Q, Hu S, Rouby JJ, *et al*. Therapeutic effects of human mesenchymal stem cell-derived microvesicles in severe pneumonia in mice. Am J Respir Crit Care Med. 2015;192(3):324-36.
3. Park J, Kim S, Lim H, Liu A, Hu S, Lee J, *et al*. Therapeutic effects of human mesenchymal stem cell microvesicles in an *ex vivo* perfused human lung injured with severe *E. coli* pneumonia. Thorax. 2019;74(1):43-50.
4. Waterman RS, Tomchuck SL, Henkle SL, Betancourt AM. A new mesenchymal stem cell (MSC) paradigm: polarization into a pro-inflammatory MSC1 or an immunosuppressive MSC2 phenotype. PLoS One. 2010;5(4):e10088.
5. Sherman BT, Hao M, Qiu J, Jiao X, Baseler MW, Lane HC, *et al*. DAVID: a web server for functional enrichment analysis and functional annotation of gene lists (2021 update). Nucleic Acids Res. 2022;50(W1):W216-21.
6. Huang DW, Sherman BT, Lempicki RA. Systematic and integrative analysis of large gene lists using DAVID bioinformatics resources. Nat Protoc. 2009;4(1):44-57.
7. Pati BA, Kurata WE, Horseman TS, Pierce LM. Antibiofilm activity of chitosan/epsilon-poly-L-lysine hydrogels in a porcine *ex vivo* skin wound polymicrobial biofilm model. Wound Repair Regen. 2021;29(2):316-26.
8. Moon AY, Bailey EJ, Polanco JA, Kurata WE, Pierce LM. Antibacterial efficacy of a chitosan-based hydrogel modified with epsilon-poly-L-lysine against *Pseudomonas aeruginosa* in a murine-infected burn wound model. Mil Med. 2023;188(Suppl 6):52-60.

9. Wu D, Qin H, Wang Z, Yu M, Liu Z, Peng H, *et al*. Bone mesenchymal stem cell-derived sEV-encapsulated thermosensitive hydrogels accelerate osteogenesis and angiogenesis by release of exosomal miR-21. Front Bioeng Biotechnol. 2022;9:829136.

10. Tao SC, Guo SC, Li M, Ke QF, Guo YP, Zhang CQ. Chitosan wound dressings incorporating exosomes derived from microrna-126-overexpressing synovium mesenchymal stem cells provide sustained release of exosomes and heal full-thickness skin defects in a diabetic rat model. Stem Cells Transl Med. 2017;6(3):736-47.

11. Mende K, Akers KS, Tyner SD, Bennett JW, Simons MP, Blyth DM, *et al*. Multidrug-resistant and virulent organisms trauma infections: Trauma Infectious Disease Outcomes Study Initiative. Mil Med. 2022;187(Suppl 2):42-51.

12. Husain ZS, Schmid S, Lombardo N. Functional outcomes after gunshot wounds to the foot and ankle. J Foot Ankle Surg. 2016;55(6):1234-40.

13. Eschbach D, Steinfeldt T, Hildebrand F, Frink M, Schöller K, Sassen M, *et al*. A porcine polytrauma model with two different degrees of hemorrhagic shock: outcome related to trauma within the first 48 h. Eur J Med Res. 2015;20(1):73.

14. Dahlquist A, Elander Degerstedt L, von Oelreich E, Brännström A, Gustavsson J, Arborelius UP, *et al*. Blast polytrauma with hemodynamic shock, hypothermia, hypoventilation and systemic inflammatory response: description of a new porcine model. Eur J Trauma Emerg Surg. 2022;48(1):401-9.

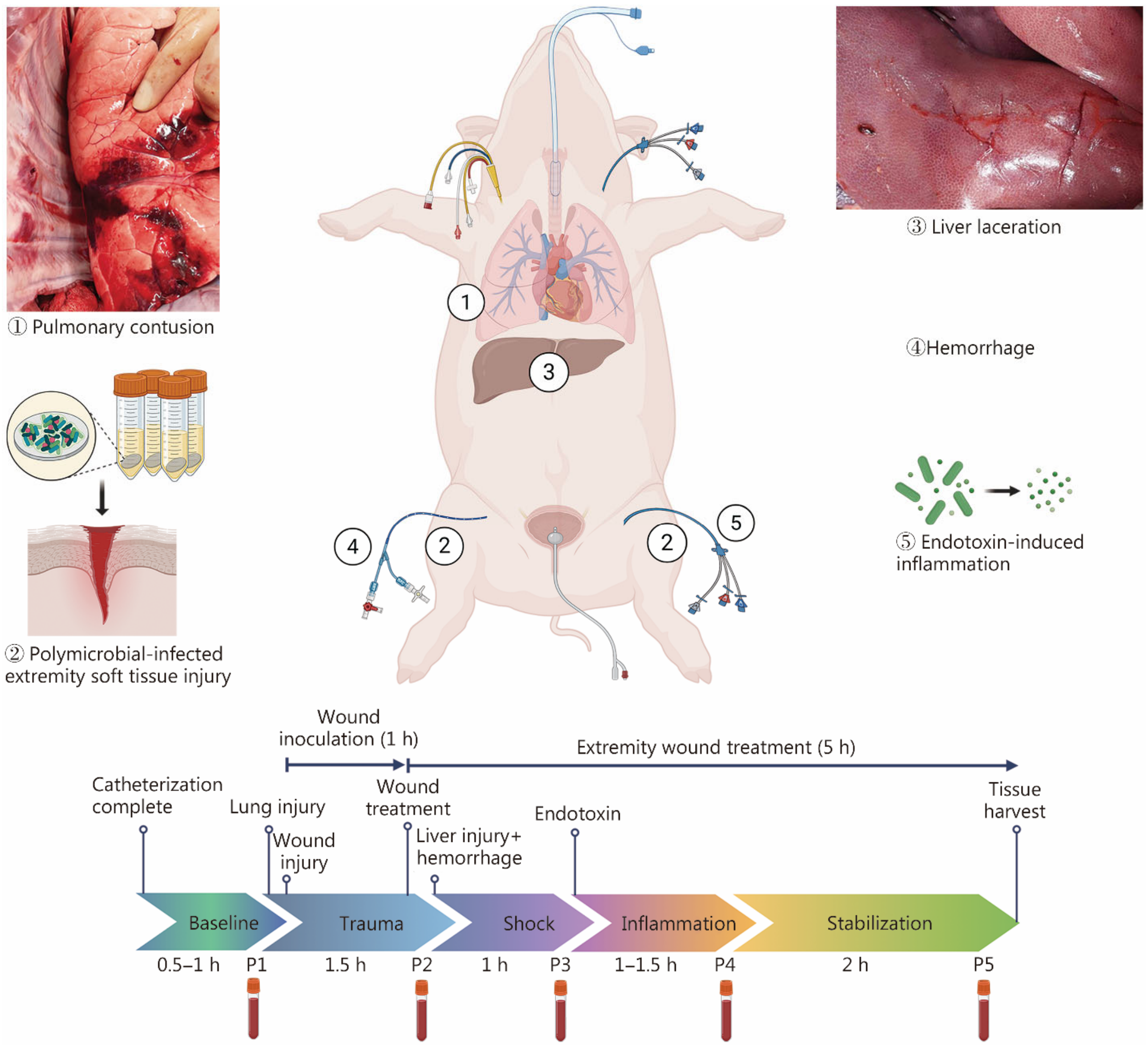

Fig. S1 Overview of the *in vivo* experimental timeline and procedures during establishment of the polytrauma state. Figure created with BioRender.com

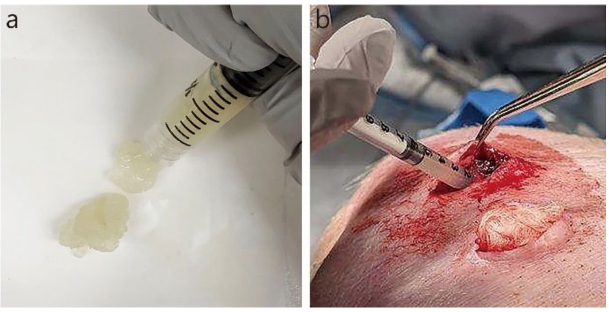

Fig. S2 Representative images of the CS/EPL hydrogel (**a**) and its application in the extremity wound cavity in the porcine polytrauma model (**b**). CS. Chitosan; EPL. Epsilon-poly-L-lysine

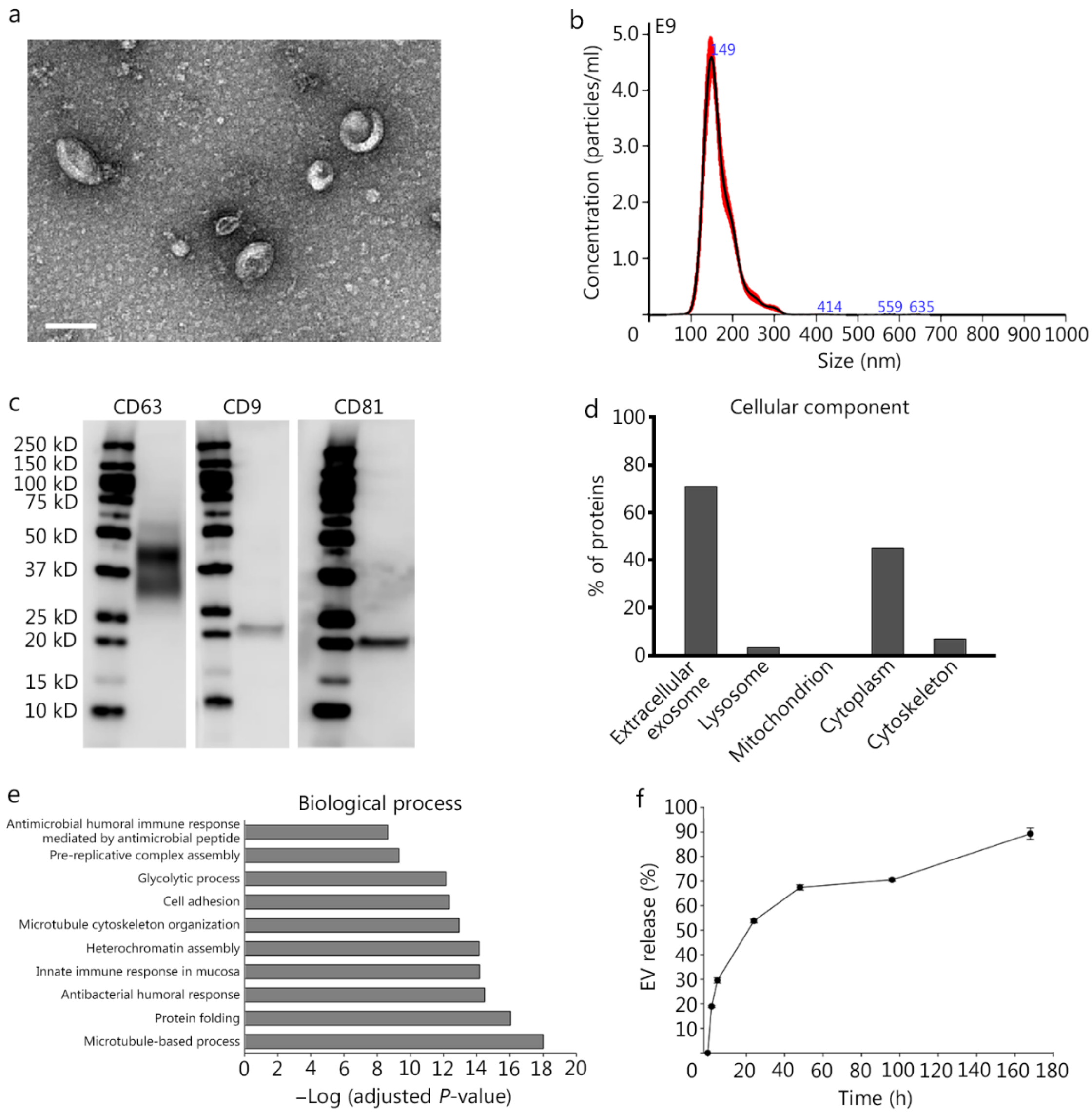

Fig. S3 Poly(I:C) EV characterization. **a** Representative transmission electron microscopy image. Scale bar=100 nm. **b** Particle size analysis of poly(I:C) EVs using nanoparticle tracking analysis. The distribution of particles occurred primarily in the 100–300 nm range (red line), with the peak concentration at 149 nm (blue number). **c** Expression of exosomal surface proteins CD63, CD9, and CD81 detected by Western blotting. Functional gene ontology enrichment analysis of poly(I:C) EV proteins identified by mass spectrometry, demonstrating distribution of proteins among cellular components (**d**) and the 10 most highly enriched biological processes (**e**). Enrichment *P*-values were adjusted by Benjamini-Hochberg False Discovery Rate Correction. **f** EV release from hydrogels in Dulbecco's phosphate-buffered saline at 37 ºC. CS. Chitosan; EV. Extracellular vesicle; Poly(I:C). Polyinosinic-polycytidylic acid

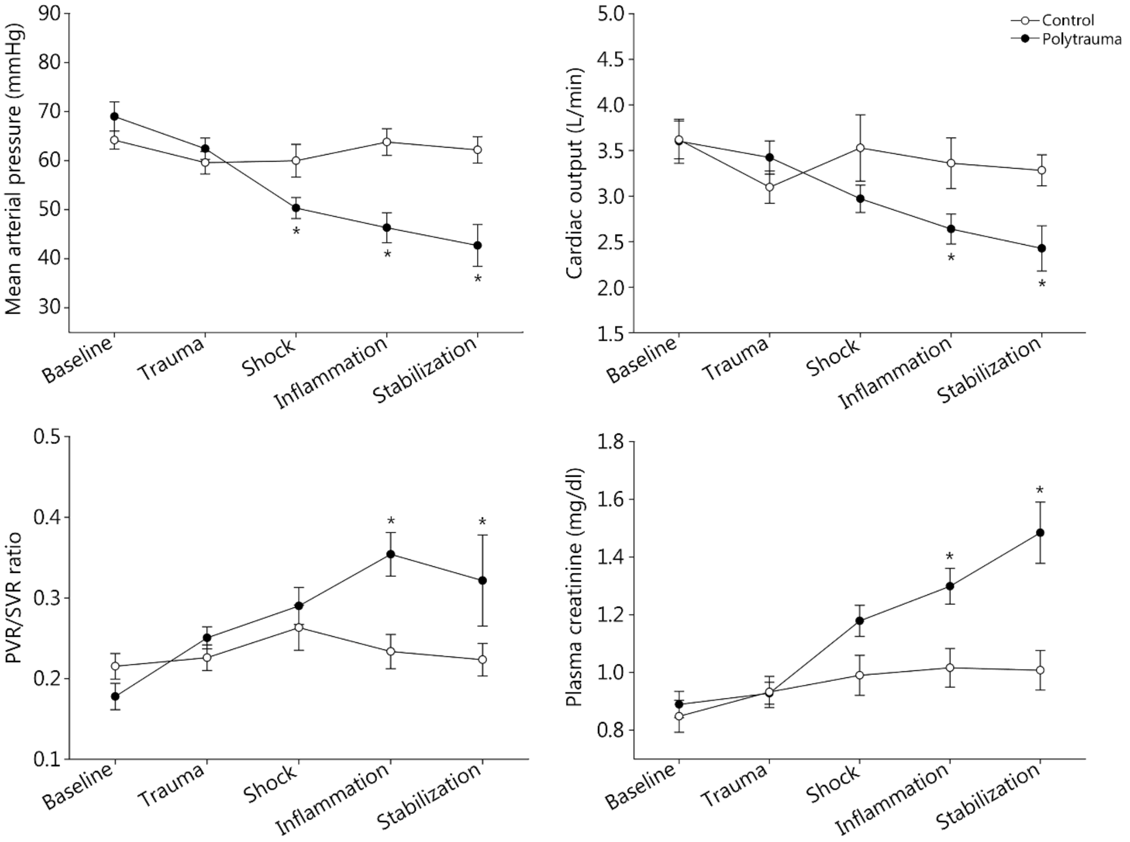

Fig. S4 Hemodynamics and plasma creatinine levels in polytrauma and control pigs, including mean arterial pressure, cardiac output, PVR/SVR ratio, and plasma creatinine. For each figure, 9 polytrauma pigs and 5 control pigs were analyzed. $^{*}P<0.05$ polytrauma vs. control, two-way repeated measures analysis of variance followed by post hoc multiple comparisons using a Bonferroni *t*-test. PVR/SVR. Pulmonary vascular resistance to systemic vascular resistance

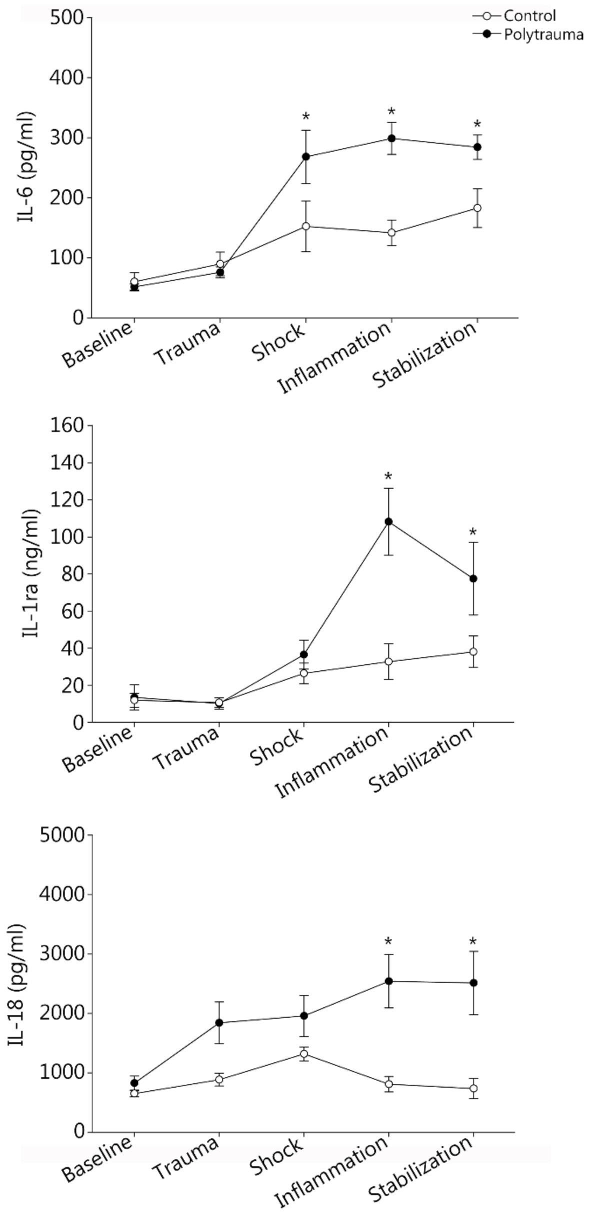

Fig. S5 Elevation of systemic inflammatory cytokine (IL-6, IL-1ra, and IL-18) levels in polytrauma. For each figure, 9 polytrauma pigs and 5 control pigs were analyzed. $^{*}P<0.05$ polytrauma vs. control, two-way repeated measures analysis of variance followed by post hoc multiple comparisons using a Bonferroni *t*-test. IL. Interleukin; IL-1ra. IL-1 receptor antagonist

Table S1 Release kinetics of extracellular vesicles (EVs) from hydrogels

Time (h)	EV release (%)	EV protein in supernatants (μg)
0	0	0
2	18.16	5.52
2	19.41	5.90
2	19.24	5.85
5	30.59	9.30
5	30.79	9.36
5	27.30	8.30
24	53.39	16.23
24	55.26	16.80
24	52.66	16.01
48	65.72	19.98
48	67.11	20.40
48	69.44	21.11
96	71.32	21.68
96	69.31	21.07
96	70.99	21.58
168	85.23	25.91
168	89.28	27.14
168	93.45	28.41
